# Design and Evaluation of Liposomal Formulation of Pilocarpine Nitrate

**DOI:** 10.4103/0250-474X.65014

**Published:** 2010

**Authors:** S. Rathod, S. G. Deshpande

**Affiliations:** C. U. Shah College of Pharmacy, Santacruz (W), Mumbai-400 049, India

**Keywords:** Intraocular pressure, liposomes, lyophilization, multilamellar vesicles, pilocarpine nitrate, thin layer film hydration

## Abstract

Prolonged release drug delivery system of pilocarpine nitrate was made by optimizing thin layer film hydration method. Egg phosphatidylcholine and cholesterol were used to make multilamellar vesicles. Effects of charges over the vesicles were studied by incorporating dicetylphosphate and stearylamine. Various factors, which may affect the size, shape, encapsulation efficiency and release rate, were studied. Liposomes in the size range 0.2 to 1 µm were obtained by optimizing the process. Encapsulation efficiency of neutral, positive and negatively charged liposomes were found to be 32.5, 35.4 and 34.2 percent, respectively. In vitro drug release rate was studied on specially designed model. Biological response in terms of reduction in intraocular pressure was observed on rabbit eyes. Pilocarpine nitrate liposomes were lyophilized and stability studies were conducted.

The importance of liposomes as drug delivery vehicle is now becoming well established. This applies particularly to the ability of liposomes to buffer the toxicity of entrapped drugs while maintaining efficacy[1], some areas in which liposomes display therapeutic promise are as carriers for anticancer agents[2–4], antiparasitic[5], antibacterial[6], antifungal drugs[7], antiviral[8,9] and ocular liposomes[10–12]. These finding makes it increasingly necessary to develop liposomes to satisfy pharmaceutical considerations. The inherent demands include inexpensive material, straightforward and rapid method of generating liposomes, homogeneous and reproducible size distribution and efficient technique for loading liposomes. In addition, the final liposomal formulation must be highly stable, as both the retention of entrapped drug as well as chemical and dimensional stability of the liposome themselves.

The behavior of liposomes *in vivo*[13] is strongly dependant upon vesicle size, lipid composition and lipid dose. In the absence of cholesterol, liposomes usually leak substantially when introduced intravenously. Most liposomes introduced i.v. is eventually cleared by the phagocytic cells of reticuloendothelial system (RES) that usually result in localization in the liver and spleen[14]. The rate of such clearance, as well as the resulting distribution, can be influenced by liposome size. Smaller vesicles are cleared more slowly than their larger counterparts (for the same lipid dose) and are less rapidly sequestered by the liver.

The circulation time is also sensitive to lipid dose; higher doses lead to longer circulation times. These effects are presumably related to saturation of RES at higher dose[15]. Other factors modulating vesicles longevity include the charge of the vesicle; vesicles containing negative charged lipids are cleared more rapidly than neutral or positively charged systems.

Multilamellar vesicles (MLVs) were prepared using thin lipid film hydration method. Various factors such as phosphatidylcholine and cholesterol ratio, lipid and drug ratio, incorporation of charged species and pH etc. were studied which may affect the size, shape and incorporation efficiency of liposomes. Liposomes were evaluated by optical microscope as well as transmission electron microscope. Analysis of drug content was carried on UV spectrophotometer. *In vitro* release rate studies were conducted on specially designed *In vitro* model and *in vivo* studies of liposomes were performed on albino rabbit eyes.

## MATERIALS AND METHODS

Pilocarpine nitrate (PN) was a gift sample from JT Baker Chemicals Co., (Philipsburg NJ), and analyzed in our laboratory. Phosphatidylcholine, cholesterol, dicetylphosphate and stearylamine were purchased from Sigma chemicals U.S. All other chemicals and reagents used were of analytical grade.

### Preparation of MLVs using thin lipid film method:

Stock solution containing phosphatidylcholine and cholesterol in 10:4 molar ratios were prepared in chloroform. Appropriate volume of these solution and 10 g glass beads were transferred to a 250 ml round bottom flask and attached to the rotary vacuum evaporator. The flask was kept immersed in a thermostat water bath, with the temperature set at 30° and rotated at about 100 rpm. Process was allowed to continue till all the liquid had evaporated from the solution and a dry lipid film had deposited on the wall of the flask. Flask was rotated under vacuum for another 15 min and then flushed with nitrogen to remove the last traces of solvent. Aqueous phase (5 ml containing drug) was added to the flask and the flask was rotated with the same speed as before for 30 min or until all the lipid had been removed from the wall of the flask. The suspension was allowed to stand for an optimized period of 2 h at room temperature in order to complete the hydration.

### Incorporation of charged species:

The inclusion of negatively charged lipid such as dicetylphosphate or positively charged surfactant such as stearyl amine tend to increase the interlamellar repeat distances between successive bilayers in the MLV, swelling the structure with the greatest proportion of the aqueous phase. These effects lead to a greater overall entrapped volume. Hence two batches of liposomes were prepared containing phosphatidylcholine, cholesterol, stearylamine and phosphatidylcholine, cholesterol and dicetylphosphate in the ratio 10:4:1 and percent drug incorporation was calculated. Antioxidant such as 1 mol % α-tocopherol[16] was used to prevent peroxidation[17–19] of lipid during sonication of MLVs.

### Size reduction of MLVs system:

Vibronics-250W probe type ultrasonicator was used for size reduction of MLV dispersion[20,21]. Sample (5 ml) was placed in a 50 ml beaker and the probe was dipped into it. The process was carried out at low temperature using ice bath. Total ultrasonication period was 3 min including intermittant stoppage of 30 s.

### Separation of non-entrapped drug:

Sample (4 ml) was placed in ultracentrifuge tubes (cooling centrifuge, Remi Instruments, Mumbai) at 20,000 rpm. Ice cold water was added to enhance centrifugation and mixture was centrifuged for 20 min. Solid particles left in the tube was collected and suitable volume of aqueous medium was added. Nitrogen gas was flushed to avoid peroxidation of lipids.

After optimization of process variables, following batches were prepared. SL-1 (phosphatidyl choline:cholesterol) 10:4, SL-2 (phophatidyl choline:cholesterol:dicetylphosphate) 10:4:1, SL-3 (phophatidyl choline:cholesterol:stearyl amine) 10:4:1.

### Microscopy:

**Fig. 1:**
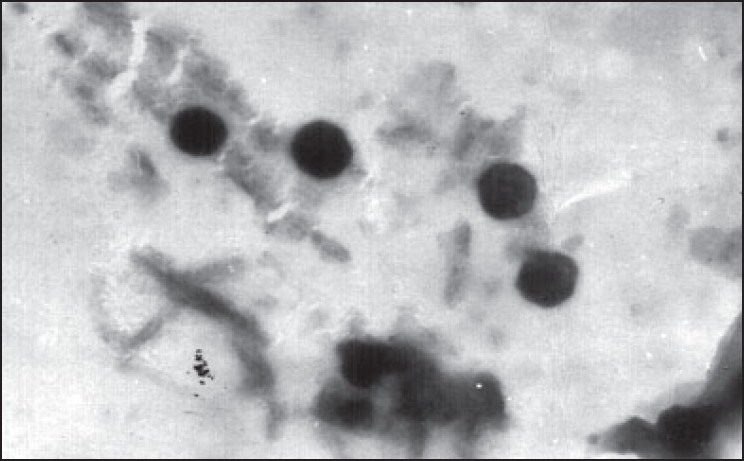
TEM Photograph of Liposome Liposomes were stained by 1.5% w/v solution of Phosphotungustic acid magnification 10000x

Every sample prepared was examined under optical microscope using oil immersion lens. Samples showing fragments of non-dispersed lipid film or PN precipitates were discarded. Electron microscopic studies were carried out on transmission electron microscope (model 100S, Geol Ltd. Tokyo, Japan). Grids precoated with colophony resins were used. Liposome sample was applied to the grid and kept for settling for 5 min. The grid was then coated with 1.5% phosphotugustate and dried at room temperature. The same was examined under transmission electron microscope and photographs were taken (fig. 1).

### Drug entrapment studies:

To aliquots of liposome sample (0.5 ml), 5 ml of 10% sodium laurylsulphate (SLS) was added and the volume was made up to 50 ml. The sample was warmed on water bath at 70° for 30 min; similarly a blank solution in 50 ml was prepared. This procedure was employed for the analysis of PN in various batches. Drug concentration was estimated by measuring the absorbance at 215 nm using spectrophotometer with reference to the blank solution prepared (Table 1).

**TABLE 1:** PERCENTAGE DRUG ENTRAPPED IN VARIOUS BATCHES

Composition	Ratio	Lipid/drug (mol/mol)	Entrapment efficiency %
PC:C	10:2	15:01	15.0
PC:C	10:4	15:01	32.5
PC:C	10.8	15.1	12.0
PC:C	10:4	15:0.5	35.8
PC:C	10:4	15:2.0	18.01
PC:C:DCP	10:4:1	15:1.0	34.2
PC:C:SA	10:4:1	15:1.0	35.4

PC is phosphatidylcholine, C is cholesterol, SA is stearyl amine and DCP is dicetyl phosphate

### In vitro drug release:

Drug release was determined with the help of modified USP XXI dissolution rate model. The model comprises of a beaker (250 ml) and a plastic tube of diameter 17.5 mm opened from both the ends. The tube was tied at one end with treated cellophane membrane and dipped into the beaker containing dissolution medium. The beaker was filled with 90 ml phosphate buffer (pH 7.4) and temperature was maintained at 37±1°. Liposomic preparation (10 ml) was added in the tube and a paddle type stirrer was attached in the center of the beaker. The speed was maintained at 100 rpm dissolution sample (5 ml) were withdrawn at 0.5, 1, 2, 4, 6 and 8 h and analyzed spectrophotometrically. Results were tabulated and graph was plotted as percentage drug release versus time for all three formulations (Table 2).

**TABLE 2:** *IN VITRO* DISSOLUTION RATE STUDIES OF PN LIPOSOMES

Time (h)	Formulation (Mean±SD)		
	LS-1	LS-2	LS-3
0.5	16.71±0.120	18.70±0.706	21.15±0.615
1.0	29.06±1.105	32.15±0.889	34.58±0.629
2.0	40.02±1.138	44.12±0.997	45.78±1.14
4.0	46.82±0.495	52.16±1.035	58.48±0.956
6.0	51.24±0.279	58.86±0.487	65.14±1.055
8.0	52.77±0.462	62.78±0.471	68.22±1.253

Formulation LS-1 contained phosphatidylcholine:cholesterol in the ration of 10:4; LS-2 contained phosphatidylcholine:cholesterol:dicetyl phosphate in the ratio of 10:4:1 and LS-3 phosphatidylcholine: cholesterol:stearylamine in the ratio of 10:4:1.

### Biological studies:

**Fig. 2:**
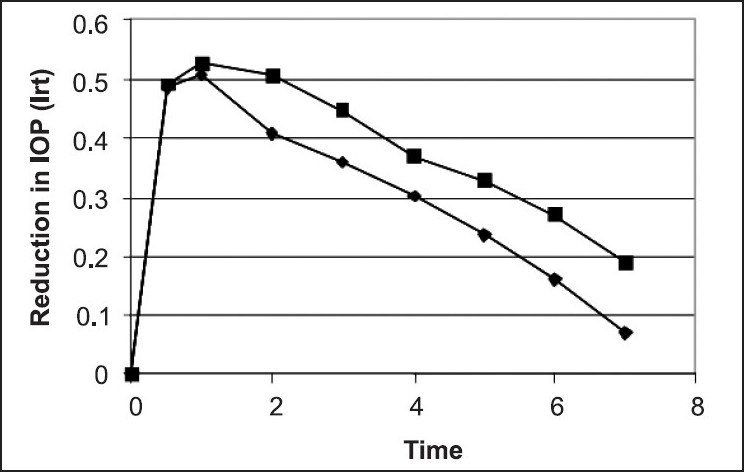
Biological response of charged liposomes Positively charged (₠) and negatively charged (♦) Liposomes

Two batches of liposomes viz. LS-2 and LS-3 were selected for animal studies. Studies were carried out in 4 albino rabbit eyes. Liposomes (50 µl) were instilled in lower cul-de-sac of one eye and the same amount of the normal saline in the other. Intraocular pressure (IOP) was measured periodically and observations were recorded in Tables 3 and 4. The response was recorded as I_rt_ = I_0_ - I_t_ / I_0_ were I_0_ is the initial IOP and I_t_ = IOP at time t (fig. 2). Marketed solutions (1, 2, and 4 % PN w/v) were also studied in the similar fashion and compared with PN liposomes (Table 5).

**TABLE 3:** BIOLOGICAL RESPONSE OF LIPOSOMES (LS-2)

Time (h)	Reduction in intra ocular pressure (Irt)				
	I	II	III	IV	Mean±SD
0.5	0.473	0.500	0.473	0.500	0.486±0.013
1	0.526	0.443	0.526	0.537	0.508±0.037
2	0.446	0.386	0.419	0.386	0.409±0.025
3	0.419	0.330	0.366	0.320	0.358±0.038
4	0.357	0.303	0.303	0.245	0.302±0.039
5	0.303	0.109	0.241	0.198	0.235±0.043
6	0.196	0.150	0.151	0.150	0.161±0.019
7	0.053	0.103	0.089	0.037	0.070±0.026

I_rt_ = I_0_ -I_t_/I_0_, where I_0_ is the initial intra ocular pressure (IOP) and I_t_ is the IOP at time t.

### Lyophilization:

Lyophilization[22–24] was carried out on an Edwards's freez-dryer. Mannitol (1 ml liposomes with 3 g mannitol) was used as cryoprotectant. Freeze dried liposomes and non lyophilized liposomes were kept for stability testing at 4°. Characteristic such as size, shape, drug content and release rate of freshly prepared and lyophilized liposomes were carried out (Table 6).

## RESULTS AND DISCUSSION

The aim of the use of the drug loaded liposome in ocular formulation is to increase the availability of the encapsulated drug to the eye in terms of both uptake and residence time. To achieve this goal, liposomes with positive and negative surface charges were prepared using negatively and positively charged components and evaluated. Thin lipid film-hydration method was used to prepare liposomes.

Process was optimized for solvent selection, lipid:lipid ratio, lipid:drug ratio, buffer system, pH, charged species added. In selection of solvent system, chloroform alone and mixture of chloroform and methanol (in ratio 2:1 and 1:1) were tried for lipid dissolution and film formation. Inclusion of methanol resulted in precipitation of some part of phospholipid before drying and/ or non-uniform film; hence chloroform alone was used. In the absence of glass beads, the lipid film formed on the wall of the flask was thick. It took longer time for hydration and fragments of non-dispersed films were formed. Hence glass beads (10g) were added to increase the surface area available for deposition of the film. Phosphatidylcholine and cholesterol in the molar ratio 10:2, 10:4 and 10:8 were tried to form liposomes. All the three batches were seen under the microscope and drug entrapment was calculated. 10:2 and 10:8 molar ratio showed some fragments of nondispersed lipids. At 10:4 ratio liposomes formed were uniform and entrapment efficiency was more (Table 1). Hence this ratio was selected for further batches.

Phospholipid:drug molar ratio was selected on the basis that lipid bilayer can intercalate drug to a maximum extent of 10% by molar ratio. On weight basis it becomes 15:1 phospholipids to drug ratio. Thus dispersion containing different molar proportions of drug was prepared (15:0.5, 15:1.0, and 15:2.0). Pilocarpine nitrate added in concentration higher than 15:1 invariably precipitated out in the system. Therefore, in all the studies, concentration of lipid and PN was kept 15:1. Drug was dispersed in buffers of different pH viz. 6.0, 6.6 7.0, 7.4 and 8.0 phosphate buffer. Buffer of pH 6.0 and 6.7 showed poor swelling of lipids with fragments of nondispersed film whereas normal saline containing sodium bicarbonate (pH 7.4) resulted in well shaped liposomes. This swelling process depends on the pH of aqueous medium. This particular lipid composition showed better swelling properties in pH range above 7. Aqueous phase of pH 7.4 was selected for the study at which drug exists in both ionized and unionized form.

Size reduction of MLV system was attempted by ultrasonication. Ice was used to lower the temperature during sonication. For determination of total drug content, the entrapped drug should be made free from liposomes. This was achieved by the addition of alcohol or detergent. Addition of SLS at higher concentration (10%) yielded a clear solution on warming at (60-70°) and did not absorb at 215 nm in UV spectrophotometer.

Optical microscopy was found to be the fast and useful method for preliminary experiment. Transmission electron microscopy enabled the determination of particle size of liposomes formed. The method for the preparation of the grid was standardized. The successes of the preparation of the grid depended on setting of the liposome particle on the coated grid surface. Settling time was kept high and a very small droplet was applied to the grid. Scanning of the grids prepared in such a manner showed a heterogeneous dispersion (0.187, 0.320, 0.487, 0.833, 1.5, 1.16 and 1.0). Photomicrographs of liposomes were taken at 10 000 X (fig. 1).

Surface potential can play an important role in the behavior of liposomes *in vivo* and *In vitro*. In general charged liposomes were more stable against aggregation and fusion than uncharged liposomes. However physically stable neutral liposomes have been described. The inclusion of negatively charged lipid such as stearylamine tends to increase the entrapped volume. (Table 1) shows the percent drug entrapment in various liposome formulations. (Table 2) shows the release of pilocarpine nitrate from neutral, negative and positively charged liposomes. The release kinetics was found to be first order initially followed by mixed order. More drug was released from charged liposomes than the neutral liposomes (Tables 3 and 4, fig. 2). A prolonged duration of response was observed during *in vivo* studies of liposome formulation. Positively charged liposome showed greater duration of action (DR 468 min) and AUC (23.5) compared to negatively charged liposomes (DR 396 min) and AUC (20.8) (Table 5). The results obtained are in accordance with the results obtained by Schaeffer[25]. The reason may be the surface of the epithelial cell membrane is slightly negatively charged therefore, positively charged liposomes interact intensively with the surface. Weissmann[26] studied the atropine base entrapped in MLVs with a positive surface charge showed a prolonged effect up to 12 h whereas in solution form, the pupil dilation lasted for 7 h. MLVs with neutral and negative charges maintained the effect for 9 h. These studies demonstrated the importance of the liposomal charge in vesicle retention.

**TABLE 4:** BIOLOGICAL RESPONSE OF LIPOSOMES (LS-3)

Time (h)	Reduction in intra ocular pressure (Irt)				
	I	II	III	IV	Mean±SD
0.5	0.554	0.424	0.526	0.471	0.491±0.047
1	0.544	0.500	0.562	0.500	0.526±0.027
2	0.526	0.490	0.529	0.490	0.508±0.018
3	0.369	0.443	0.482	0.386	0.445±0.036
4	0.312	0.292	0.419	0.292	0.328±0.052
5	0.303	0.109	0.241	0.198	0.235±0.043
6	0.267	0.245	0.303	0.264	0.269±0.020
7	0.160	0.198	0.198	0.198	0.188±0.016
8	0.107	0.037	0.089	0.103	0.084±0.027

I_rt_ = I_0_ -I_T_/I_0_, where I_0_ is the initial intra ocular pressure (IOP) and I_t_ is the IOP at time t.

**TABLE 5:** COMPARATIVE EVALUATION OF VARIOUS FORMULATIONS CONTAINING 1% PN

Formulation	TM (min)	PT (min)	Irt (max)	DR (min)	AUC
Positively charged liposomes	12	60	0.53	468	23.5
Negatively charged liposomes	12	60	0.505	396	20.8
PN solution	6	30	0.431	155	06.5
2% Carbopol gel containing PN	6	30	0.468	240	14.21

TM- time required to achieve significant reduction in IOP (Irt = 0.1), PT - time required to achieve maximum reduction in IOP, Irt (max) - maximum reduction in intraocular pressure, DR - duration of miotic response, AUC - area under curve

**TABLE 6:** COMPARATIVE STUDIES OF FRESHLY PREPARED AND LYOPHILISED LIPOSOMES

Characteristics	Freshly prepared liposomes	Lyophilised liposomes
Shape	Spherical	Spherical
Colour	White	White
Odour	Odourless	Odourless
Size	Below 1 µ	Below 1 µ
Entrapped drug	35.48%	35.2%
Release Rate on		
Day 1	68.20%	68.05%
Day 7	68.09%	67.75%
Day 14	68.22%	67.39%

The characteristics of lyophilized and non-lyophilized liposomes were examined. Lyophilization of liposomes had no substantial effect on size and shape. They could be easily dispersed to give a suspension of aggregate free liposomes. The drug content of freshly prepared liposomes and lyophilized liposome were found to be the same. No difference was found in the drug release rate. Both lyophilized and freshly prepared liposomes were stored in the refrigerator at 4°. Studies revealed that non-lyophilised liposomes were stable for 6 weeks only whereas lyophilized liposomes were stable for 6 months and above. Mannitol was used in the ratio 2:1 mannitol to lipid for lyophilization, which helped in reducing the time required for the process as well as acted as cryoprotectant.

In conclusion, delivery of liposome encapsulated drugs as eye drops improved the extent of uptake and residence time compared to the free drug solution. Positively charged liposomes interact more with the corneal surface thus prolong the residence time.
